# Antimicrobial Peptides Relieve Transportation Stress in Ragdoll Cats by Regulating the Gut Microbiota

**DOI:** 10.3390/metabo13030326

**Published:** 2023-02-22

**Authors:** Shansong He, Kang Yang, Jiawei Wen, Tao Kuang, Zhihao Cao, Lingna Zhang, Sufang Han, Shiyan Jian, Xin Chen, Limeng Zhang, Jinping Deng, Baichuan Deng

**Affiliations:** 1 Guangdong Provincial Key Laboratory of Animal Nutrition Control, National Engineering Research Center for Breeding Swine Industry, College of Animal Science, South China Agricultural University, Guangzhou 510642, China; 2 School of Medicine, Foshan University, Foshan 528000, China; 3 Research Center of Pet Nutrition, Guangzhou Qingke Biotechnology Co., Ltd., Guangzhou 510642, China

**Keywords:** transportation stress, antimicrobial peptides, cats, pet food, microbiota, metabolome

## Abstract

Transportation is common in cats and often causes stress and intestinal disorders. Antimicrobial peptides (AMPs) exhibit a broad spectrum of antibacterial activity, and they may have the capacity for antioxidant and immune regulation. The objective of this study was to investigate the effects of dietary supplementation with AMPs on stress response, gut microbiota and metabolites of cats that have undergone transport stress. A total of 14 Ragdoll cats were randomly allocated into 2 treatments: basal diet (CON) and a basal diet supplemented with 0.3% AMPs. After a 6-week feeding period, all cats were transported for 3 h and, then, fed for another week. The results show that the diarrhea rate of cats was markedly reduced by supplementation with AMPs throughout the trial period (*p* < 0.05). In addition, AMPs significantly reduced serum cortisol and serum amyloid A (*p* < 0.05) and increased apolipoprotein 1 after transportation (*p* < 0.05). Moreover, AMPs reduced the level of inflammatory factors in the serum caused by transportation stress, including tumor necrosis factor-alpha (TNF-α) and interleukin-1 beta (IL-1β) (*p* < 0.05). The AMPs enhanced the activities of glutathione peroxidase (*p* < 0.01) and superoxide dismutase (*p* < 0.05). Furthermore, cats fed AMPs had higher levels of branched chain fatty acids (BCFAs) and a relative abundance of *Blautia* and a lower relative abundance of *Negativibacillus* after transportation (*p* < 0.05). The serum metabolome analysis further revealed that AMPs markedly regulated lipid metabolism by upregulating cholic acid expression. In conclusion, AMP supplementation alleviated oxidative stress and inflammatory response in transportation by regulating the gut microbiota and metabolites, thereby relieving stress-induced diarrhea and supporting gut and host health in cats.

## 1. Introduction

Cats as companion animals have become increasingly popular worldwide in recent years. According to the reports, the number of domestic cats is 58.06 million in China and 113.59 million in Europe in 2021 [1,2]. Furthermore, transportation is common for cats during vet visits or outings. Previous studies revealed that transportation can be particularly stressful due to the novel environment, engine noise and the irregular movement of the vehicle [3,4]. Therefore, reducing the stress caused by transportation has become a big concern for pet owners.

Stress is broadly defined as the physiological response when homeostasis is threatened [5]. Stress induced by transportation may result in diarrhea, weight loss, and the increased production of cell-free radicals, therefore causing oxidative stress [3,6]. Previous studies have shown that transportation stress activates the hypothalamic–pituitary–adrenal (HPA) axis and promotes the release of glucocorticoids, mainly cortisol (COR) and corticosterone [7]. At the same time, macrophages and lymphocytes release multiple cytokines (interleukins, interferons, tumor necrosis factors, etc.), among which proinflammatory cytokines stimulate the liver to synthesize acute phase proteins (APPs), such as serum amyloid A (SAA) and apolipoprotein 1 (Apo-A1) [8,9,10]. In addition, a growing number of studies have found a close link between stress and gut microbiota [11]. Transportation stress can change the intestinal flora of animals, reduce intestinal microbial richness, increase intestinal permeability [12,13], and cause gastrointestinal diseases, including diarrhea and acute intestinal infection [14].

Feline intestinal flora is mainly composed of *Firmicutes*, *Bacteroidetes*, *Actinobacteria*, and *Proteobacteria* [15]. The intestinal microbiota can regulate the host’s health by producing metabolites. Commensal bacteria in the colon use dietary fiber to synthesize short-chain fatty acids (SCFAs) to regulate immune function and intestinal mucosal barrier function [16]. *Atoposipes* and *Pseudomonas* are dominant branched-chain fatty acids (BCFAs) that produce bacteria, which can fight against inflammation and increase the expression of anti-inflammatory cytokines [17].

Antimicrobial peptides (AMPs), as a kind of polypeptide, are widely distributed in insects, amphibians and mammals. They have strong cationic properties, high stability, and broad-spectrum antibacterial activity against a variety of microorganisms (bacteria, fungi, viruses and parasites) [18,19]. Furthermore, AMPs have been shown to effectively prevent diarrhea in animals by eliminating harmful bacteria, including *Escherichia coli* (*E. coli*) [20,21]. A previous study proposed that AMPs could alleviate inflammatory bowel disease by modulating the gut microbiota in mice [22]. Furthermore, AMPs have been reported to have beneficial effects on intestinal morphology, enhancing the intestinal barrier in broilers [23]. However, few studies have investigated the effects of AMPs on domestic cats. Thus, it is of great significance to study the protection of cat health by adding AMPs to diets.

Ragdoll cats are common in pet-owning families. The objectives of this study were to determine the potential of AMPs as food additives to alleviate transportation stress. To be specific, we investigated the effects of an AMP supplement diet on the diarrhea rate, serum hormone, inflammatory factors, antioxidant indicators, fecal microbiota and metabolites of cats before and after transportation. The aim of this study is to provide a reference for the further future study studies about the significance of AMPs with respect to the intestinal health of cats.

## 2. Materials and Methods

All experimental procedures were approved by the Experiment Animal Ethics Committee of South China Agricultural University.

### 2.1. Animals and Diets

Fourteen ragdolls (four males and ten females, age 1–2 years) were allotted to two treatments (n = 7/group) on the basis of body weight (BW) and sex, including a basal diet (CON) group and a basal diet supplemented with the 0.3% AMP group. The BW of the CON group is 4.18 ± 0.84 Kg, the BW of the AMPs group is 4.08 ± 1.16 Kg, and there are no differences between the two groups (*p* > 0.05). The AMP products were purchased from Guangdong Genuizymes Animal Health Co., Ltd. (Guangzhou, China), which were isolated from chicken intestines and cultured by *Bacillus subtilis*. The dose of dietary AMPs was based on the findings of our previously unpublished data, showing that 0.3% of AMPs was effective in preventing diarrhea in cats. The composition and nutrition levels of a basal diet are shown in Table 1, which met the requirements of the National Research Council (NCR, 2006).

### 2.2. Experimental Design

This study included two periods: the feeding period and transportation period. In the feeding period, each ragdoll was raised individually in cat cages (1.1 × 0.7 × 0.55 m) in a temperature-controlled room for 42 days, and was fed at 8:30 am each day. Ragdolls had free access to fresh water. All cats were dewormed quarterly and no drugs (such as antibiotics) were given 1 month before the study. Feed intake and fecal score (FS) were recorded once a day. FS is graded on a scale from 1 to 5, where grade 1 represented dry crumbly feces, grade 2 represented dry feces with little water, grade 3 represented normal moist feces, grade 4 represented wet feces, and grade 5 represented diarrhea [24]. BW was recorded once a week.

**Figure 1:**
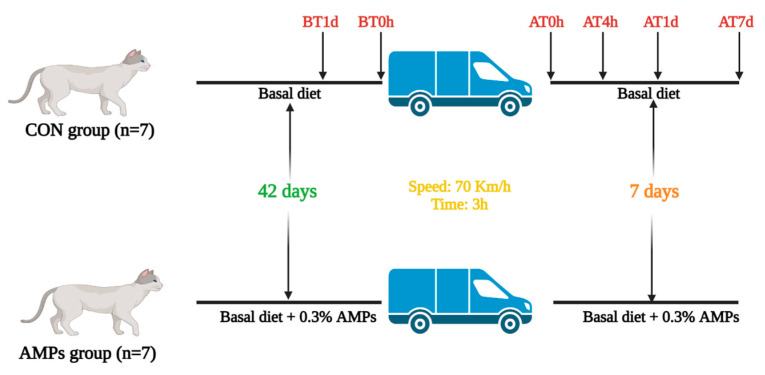
The sketch map of the study’s design. The CON group was fed a basal diet (n = 7), and the AMPs group was fed a basal diet with 0.3% AMPs (n = 7). BT1d, 1 day before transportation; BT0h, right before transportation; AT0h, right after transportation; AT4h, 4 h after transportation; AT1d, 1 day after transportation; AT7d, 7 days after transportation.

On day 43, all cats were transported back and forth from Guangzhou city to Zhaoqing city (Guangdong province, China), and were transported for 3 h with an average speed of 70 Km/h. The truck size is 5.2 × 2.2 × 2.1 m, and the temperature was 23 °C with 60% relative humidity. Transportation started at 9:20 am, and the roads include city roads and highways. After transportation, FS recordings were continued for 7 days. The study’s design is depicted in Figure 1.

### 2.3. Blood Sample Collection and Analysis

Blood samples were obtained at the time right before transportation (BT0h), right after transportation (AT0h) and then 4 h (AT4h), 1 d (AT1d) and 7 d post-transportation (AT7d). Approximately 3 mL of blood was taken into a 10 mL anticoagulant-free vacutainer tube. After holding a 45° angle for 30 min, the serum was collected by centrifugation at 3500× *g* rpm for 15 min at room temperature, and stored at −80 °C for serum biochemical parameter measurements. COR, SAA, Apo-A1, interleukin-1beta (IL-1β), tumor necrosis factor-alpha (TNF-α) and interferon-gamma (IFN-γ) were analyzed by using commercial ELISA kits (MEIMIAN, Jiangsu Meimian Industrial Co., Ltd., Yancheng, China). Serum catalase (CAT), total antioxidant capacity (T-AOC), glutathione peroxidase (GSH-Px), superoxide dismutase (SOD) and malondialdehydes (MDA) were measured using commercial kits (Nanjing Jiancheng Bioengineering Institute, Nanjing, China) according to the manufacturer’s protocol.

### 2.4. Fresh Fecal Sample Collection and Analysis

On BT1d and AT1d, cats’ fresh feces were collected within 15 min of defecation, and stored at −80 °C for further analyses. An aliquot was collected for fecal short-chain fatty acids (SCFAs) and branched-chain fatty acids (BCFAs) measurements. An aliquot was collected for microbiota measurements. Lastly, an aliquot was collected for metabolomics analysis.

#### 2.4.1. Fecal SCFAs and BCFAs Analysis

The new fecal samples gathered on BT1d and AT1d were preprocessed, and SCFAs and BCFAs were extracted according to the method described in our previous work [6]. The quantitative analysis of SCFAs and BCFAs was carried out using the GCMS-QP2020 system (Shimadzu, Kyoto, Japan). Instrumental parameters were obtained with the method established in our laboratory [6].

#### 2.4.2. 16S rRNA High-Throughput Sequencing

Fresh fecal samples were collected on BT1d and AT1d, and transported to Hangzhou Lianchuan Biotechnology Co., Ltd. (Hangzhou, China) for gut microbial analysis and sequenced on the Illumina NovaSeq platform. In brief, genome DNA was extracted using the cetyltrimethylammonium bromide method, and then the highly variable region V3-V4 of the 16S rDNA gene was amplified; the quality of the obtained PCR amplification products was monitored on 1% agarose gels. Finally, the samples were subjected to the high-throughput sequencing of 16S rRNA genes on the Illumina MiSeq platform of Lianchuan Biologicals. After the completion of sequencing, raw data were obtained. Later, the splicing of double-ended data with overlap was carried out, while high-quality clean data were obtained via the quality control and filtering of chimera. Afterward, the feature table and sequences for further analyses of diversity, species taxonomic annotation and difference analysis were obtained. Finally, the Amplicon Sequence Variant’s (ASV) feature table and sequence were obtained.

The QIIME2 process was used to analyze alpha diversity (α-diversity) and beta diversity (β-diversity), and the pictures were displayed with R software (Version 3. 5.2). Linear discriminant analysis (LDA) and effect size (LEfSe) were processed by using the LDA score of >2.5 using LEfSe software.

### 2.5. Serum Untargeted Metabolomics Analysis

The serum metabolome was assessed using an untargeted approach via UPLC-MS/MS (Thermo Fisher Scientific, Waltham, MA, USA). The sample pretreatment methods and UPLC-MS/MS analysis method were carried out as described previously [6,25], with minor modification. In brief, we removed the protein from the serum sample and then dried the supernatant for testing. Raw data were preprocessed by compound discoverer 2.1 software (Thermo Fisher Scientific). Pathway enrichment analyses were processed in MetaboAnalyst 5.0 (https://www.metaboanalyst.ca (accessed on 10 September 2022)). The visualization results of the models were obtained with MetaboAnalyst 5.0.

### 2.6. Statistical Analysis

Original data were initially processed by Excel 2019. All data were expressed as means±standard deviation and analyzed using an unpaired Student’s *t*-test in SPSS 26.0. *p* < 0.05 was defined as statistical significance, and 0.05 < *p* < 0.10 was defined as a trend of difference. Graphs were drawn using GraphPad Prism 8.0 software. Microbe were compared using LEfSe, and significant differences were denoted by an LDA score > 2.5, Kruskal–Wallis rank sum test < 0.05 and Wilcoxon rank sum test < 0.05. The differential microbial were calculated by the Wilcoxon rank sum test. The volcano plot was constructed using OmicStudio tools available at https://www.omicstudio.cn/tool (accessed on 10 September 2022). For metabolome data, an unpaired Student’s *t*-test was performed between the CON and AMPs groups, with *p* < 0.05 and VIP > 1 indicating significantly different metabolites. A clustering correlation heatmap with signs was drawn using the OmicStudio tools and Spearman’s correlation analysis (R version 3.6.1).

## 3. Results

### 3.1. Effect of AMPs on Diarrhea Rate and Serum Hormone in Ragdolls

**Figure 2:**
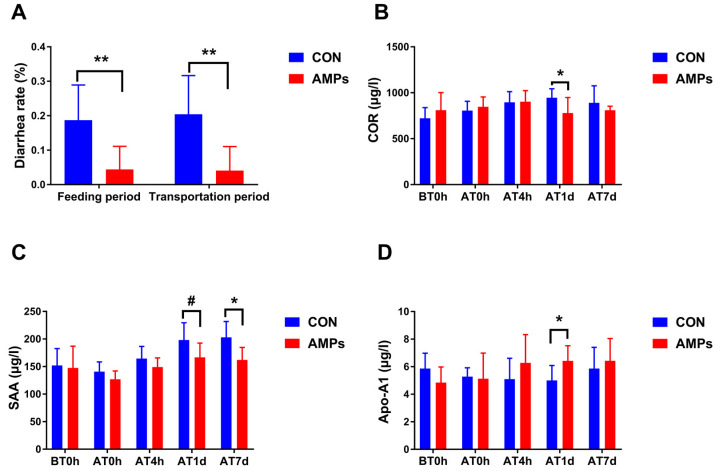
Effect of AMPs on diarrhea rate (**A**) and serum hormone (**B**–**D**) in ragdolls. Symbol (*) indicates statistically significant differences between two groups (* *p* < 0.05 and ** *p* < 0.01), and symbol (#) indicates difference tendencies (0.05 < ^#^ *p* < 0.10). BT0h, right before transportation; AT0h, right after transportation; AT4h, 4 h after transportation; AT1d, 1 day after transportation; AT7d, 7 days after transportation.

Compared with the CON group, the AMPs group had a significantly lower diarrhea rate during the feeding period and transportation period (*p* < 0.05, Figure 2A). Compared with the CON group, AMPs reduced the diarrhea rate by as much as 12.3% and 16.3% in the feeding period and transportation period, respectively. On AT1d, ragdolls fed with AMPs had a lower COR concentration (*p* < 0.05, Figure 2B). The AMPs group had a trend toward lower SAA levels on AT1d (*p* = 0.062, Figure 2C) and significantly decreased SAA levels on AT7d (*p* < 0.05, Figure 2C). Moreover, the AMPs group had a higher Apo-A1 concentration on AT1d (*p* < 0.05, Figure 2D).

### 3.2. Effect of AMPs on Serum Inflammatory Factors and Antioxidant Capacity in Ragdolls

**Figure 3:**
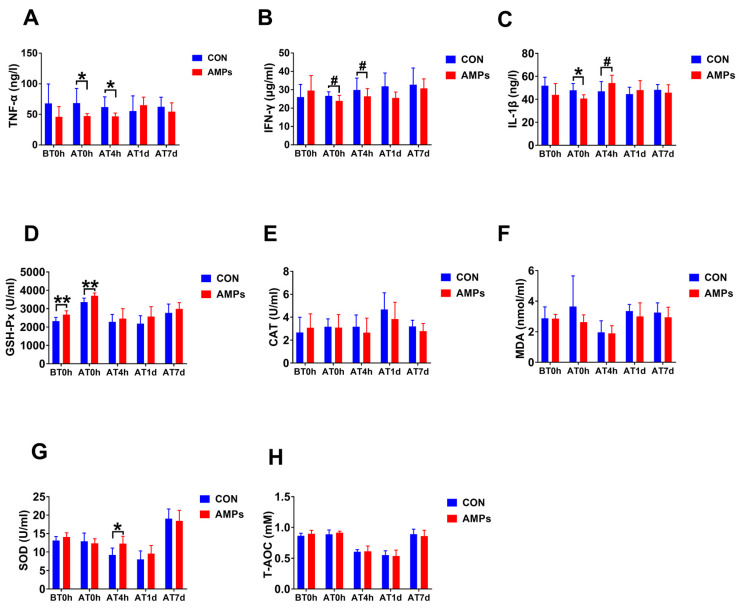
Effect of AMPs on inflammatory factors (**A**–**C**) and antioxidant capacity (**D**–**H**) in ragdolls. Symbol (*) indicates statistically significant differences between two groups (* *p* < 0.05 and ** *p* < 0.01), and symbol (#) indicates difference tendencies (0.05 < ^#^ *p* < 0.10) between two groups. BT0h, right before transportation; AT0h, right after transportation; AT4h, 4 h after transportation; AT1d, 1 day after transportation; AT7d, 7 days after transportation.

At AT0h and AT4h, the AMPs group had a decreasing TNF-α level over the CON group (*p* < 0.05, Figure 3A). Similarly, the AMPs group tended to reduce the INF-γ level at AT0h (*p* = 0.075, Figure 3B) and AT4h (*p* = 0.059, Figure 3B). Ragdolls fed with AMPs had a lower IL-1β level than the CON group at BT0h (*p* < 0.05, Figure 3C), and they exhibited an increasing trend at AT4h (*p* = 0.099, Figure 3C).

After feeding AMPs for 42 days, ragdolls had increased GSH-Px levels over the CON group before transportation (*p* < 0.01, Figure 3D). After transportation, the AMPs group had a higher GSH-Px level than the CON group at BT0h (*p* < 0.01, Figure 3D). Additionally, the AMPs group had a higher SOD level than the CON group at AT4h (*p* < 0.05, Figure 3G). Serum MDA, CAT and T-AOC levels had no obvious difference between two groups (*p* > 0.05, Figure 3E,F,H).

### 3.3. Effect of AMPs on Fecal Microbiota in Ragdolls

**Figure 4:**
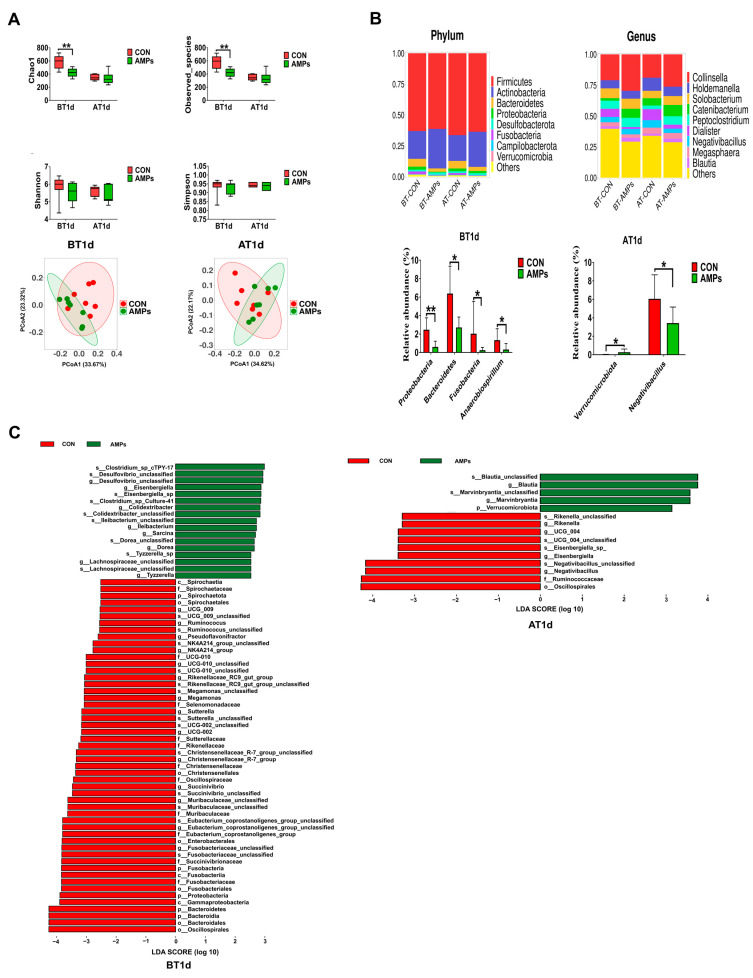
Effect of AMPs on fecal microbial. α-diversity and principal coordinate analysis (PCoA) based on weighted UniFrac distances (**A**); predominant fecal microbial communities (**B**); the linear discriminant analysis effect size (LEfSe) analysis identified gut bacterial biomarkers (**C**). Symbol (*) indicates statistically significant differences between two groups (* *p <* 0.05 and ** *p <* 0.01). BT1d, 1 day before transportation; AT1d, 1 day after transportation.

After feeding AMPs for 42 days, ragdolls had a markedly decreased chao1 index and observed_species compared with the CON group on BT1d (*p* < 0.01, Figure 4A), but there was no significant difference between two groups after transportation (*p* > 0.1, Figure 4A). Both Shannon and Simpson indices show no difference on BT1d and AT1d (*p* > 0.1, Figure 4A). From the beta diversity index based on weighted UniFrac distances, the CON group shows a trend toward significant separation relative to the AMPs group on BT1d (*p* = 0.05, Figure 4A), whereas no separation was observed between two groups on AT1d (*p* = 0.408, Figure 4A).

As shown in Figure 4B, the predominant fecal phyla included *Firmicutes*, *Actinobacteria*, *Bacteroidetes*, *Proteobacteria* and *Desulfobacterota* in ragdolls at different time points. Furthermore, the most abundant genera were *Collinsella*, *Holdemanella*, *Solobacterium*, *Catenibacterium*, *Peptoclostridium*, *Dialister*, *Negativibacillus*, *Megasphaera* and *Blautia*. Wilcoxon test results revealed that cats fed withAMPs had lower Proteobacteria (*p* < 0.01), *Bacteroidetes* (*p* < 0.05), *Fusobacteria* (*p* < 0.05) and *Anaerostipes* (*p* < 0.05) compared with the CON group on BT1d. Furthermore, the AMPs group had a higher relative abundance of *Verrucomicrobiota* (*p* < 0.05) and a lower relative abundance of *Negativibacillus* than the CON group on AT1d (*p* < 0.05).

We used the LEfSe analysis to further distinguish differential taxon abundances. On BT1d, the LEfSe analysis indicated that *Bacteroidetes* and *Proteobacteria* dominated in the CON group, while *Eisenbergiella* was the most abundant in the AMPs group. Furthermore, *Negativibacillus* was the predominant bacterial strain in the CON group on AT1d, whereas *Blautia* numbered the highest in the AMPs group.

### 3.4. Effect of AMPs on Fecal SCFAs and BCFAs in Ragdolls

**Figure 5:**
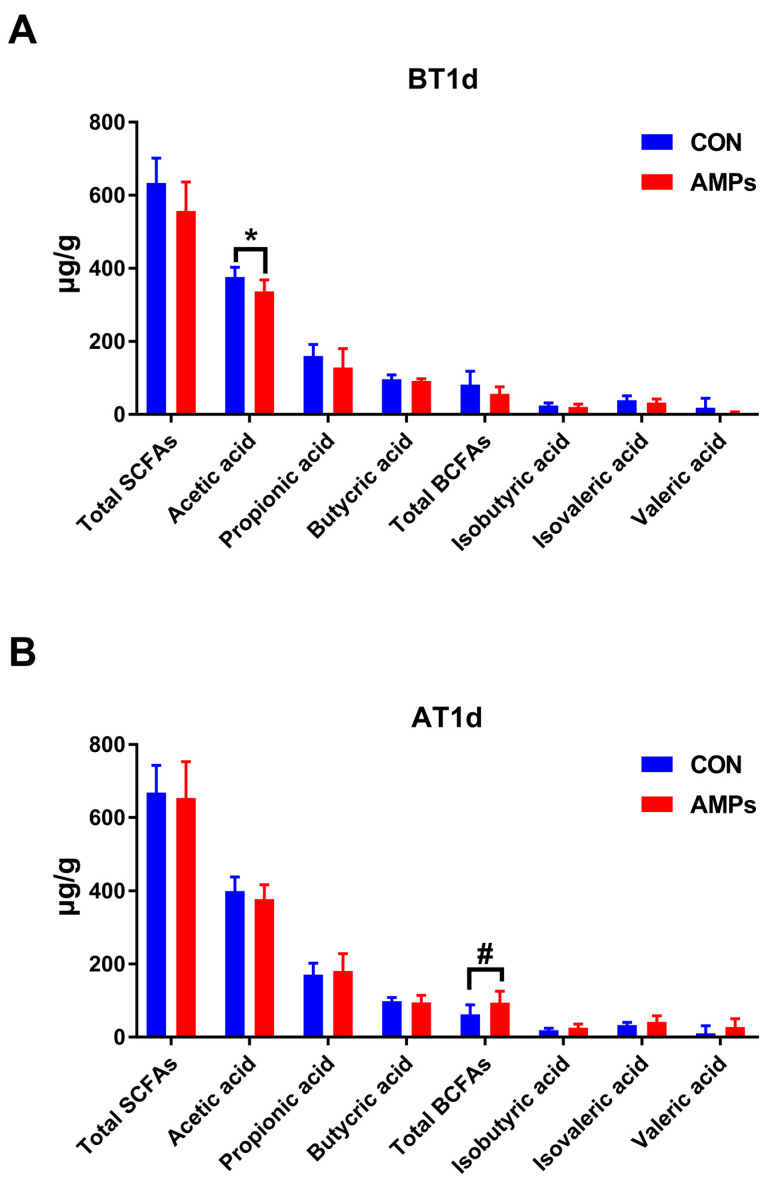
Effect of AMPs on the production of SCFAs (**A**) and BCFAs (**B**) in ragdolls. Symbol (*) indicates statistically significant differences between two groups (* *p <* 0.05), and symbol (#) indicates difference tendencies (0.05 < ^#^ *p <* 0.10) between two groups. BT1d, 1 day before transportation; AT1d, 1 day after transportation.

As shown in Figure 5, after feeding with AMPs for 42 days, ragdolls had a lower acetic acid level compared with the CON group on BT1d (*p* < 0.05, Figure 5A). After transportation, the AMPs group had an increasing trend toward total BFCAs compared with the CON group (*p* = 0.055, Figure 5B).

### 3.5. Effect of AMPs on Serum Metabolic Profiles in Ragdolls

**Figure 6:**
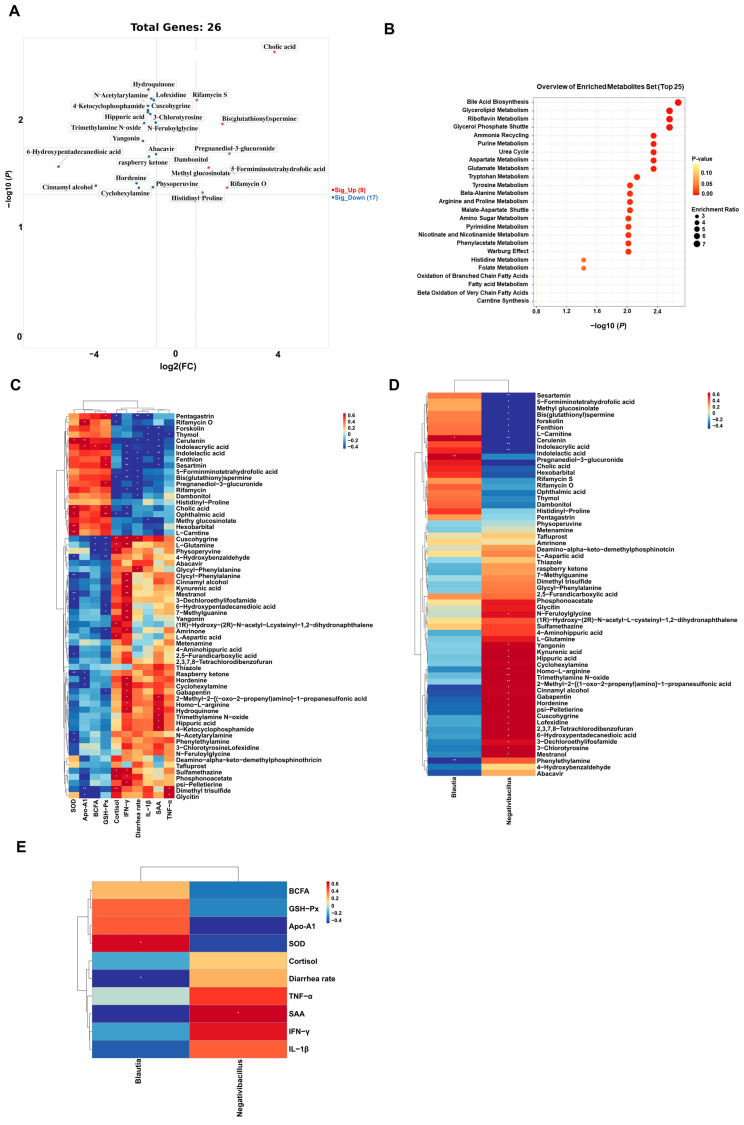
Serum metabolic profiles of cats, and Spearman’s correlation analysis. Volcano plot (**A**), KEGG metabolic pathways enrichment analysis (**B**), heatmaps of Spearman’s correlation analysis between (**C**) differential serum metabolites and serum/fecal parameters, (**D**) differential serum metabolites and fecal microbiota, (**E**) differential fecal microbiota and serum/fecal parameters. Red and blue boxes represent positive and negative correlations.

In our study, a total of 366 serum metabolites were monitored at all stages. Due to the fact that very few differential serum metabolites were identified on BT1d, thus, we didn’t obtain the data onserum metabolites on BT1d. Differential serum metabolites between the CON and AMPs groups were screened out using the standard of VIP > 1 and *p* < 0.05 on AT1d. The volcano plot (fold change >2/<0.5) shows that there were 16 metabolites upregulated and 9 metabolites downregulated in the AMPs group compared with the CON group. The primary significant metabolites were cholic acid, rifamycin S, bis (glutathionyl) spermine, hydroquinone, lofexidine and cuscohygrine (Figure 6A). To determine the functions of these differential metabolites, we performed the KEGG enrichment analysis; the results indicated that bile acid biosynthesis, glycerolipid metabolism, riboflavin metabolism and glycerol phosphate shuttle were significantly enriched (Figure 6B).

To further clarify the association among gut microbiota, serum metabolites and differential parameters, we performed Spearman’s correlation analysis among differential serum metabolites, the differential serum/fecal parameters and the differential fecal microbiota. The results are visualized as heatmaps in Figure 6. As shown in Figure 6C, SOD was positively associated with hexobarbital, cholic acid and L-carnitine but negatively associated with hordenine, cinnamyl alcohol and 3-dechloroethylifosfamide. Apo-A1 was positively associated with cerulenin but negatively associated with dimethyl trisulfide. GSH-Px was positively associated with ophthalmic acid, but negatively associated with 4-hydroxybenzaldehyde and cuscohygrine. SAS was negatively associated with indoleacrylic acid, indolelactic acid and forskolin. Cortisol was positively associated with dimethyl trisulfide and cuscohygrine. IFN-γ was positively associated with mestranol, cyclohexylamine, cuscohygrine, kynurenic acid, L-glutamine, (1R)-hydroxy-(2R)-n-acetyl-l-cysteinyl-1,2-dihydronaphthalene, yangonin, cinnamyl alcohol and 3-dechloroethylifosfamide, but it was negatively associated with indolelactic acid, fenthion, sesartemin, bis(glutathionyl)spermine and ophthalmic acid. As shown in Figure 6D, *Blautia* was positively associated with pregnanediol-3-glucuronide but negatively associated with phenylethylamine. *Negativibacillus* was positively associated with cyclohexylamine, homo-L-arginine and trimethylamine n-oxide, but it was negatively associated with sesartemin, cerulenin, indolelactic acid and indoleacrylic acid. As shown in Figure 6E, *Blautia* was positively associated with SOD and the diarrhea rate, and *Negativibacillus* was positively associated with SAA.

## 4. Discussion

### 4.1. AMPs Relieve Transportation Stress in Ragdoll Cats

In this study, we have demonstrated that AMPs could alleviate diarrhea symptoms, suppress inflammation and enhance antioxidant capacities in ragdolls under road transportation. Feng et al. confirmed that the antimicrobial peptide cathelicidin-BF had therapeutic effects for post-weaning diarrhea in piglets by improving immune response and attenuating intestinal inflammation [21]. A previous study conducted by Wu et al. also found that AMPs treatments reduced the incidence of diarrhea in weaned piglets challenged with *E. coli* [26]. Similar results were also obtained in our study. Our results suggested that dietary AMPs supplementation reduced diarrhea in cats before and after transportation.

As many studies indicated, transportation stress activates the HPA axis and promotes the release of glucocorticoids, including COR [27]. Our study shows an increasing serum COR level after transportation, indicating that cats have suffered from stress. We found cats fed with 0.3% AMPs had a lower serum COR level on AT1d. Furthermore, stress can lead to an increase in acute phase protein (APP). According to the increase or decrease in concentration, APP can be divided into positive acute proteins and negative acute proteins. Positive acute proteins mainly include SAA and C-reactive proteins, and negative acute proteins mainly include Apo-A1 and albumin [10,28]. Increasing serum SAA and decreasing Apo-A1 levels were observed in this study. Nevertheless, AMPs reduced SAA levels on AT1d and AT7d and increased Apo-A1 levels at AT4h, indicating that AMPs have great potential toward relieving stress.

Previous studies revealed that AMPs not only have antimicrobial activities but also play an important role in innate immunity [29]. AMPs maintain the homeostasis of the immune microenvironment by regulating cytokines such as interleukins, tumor necrosis factors, interferons and the activities of immune cells (e.g., dendritic cells, monocytes and mast cells) [18]. Sorrells et al. and Khan et al. observed that acute stress could enhance the body’s immune function [30,31]. Zhai et al. found that AMPs could downregulate the mRNA expression of TNF-α, IL-6 and IL-8 [32]. Similarly, our results showed that AMPs effectively alleviated inflammatory responses in cats by downregulating the release of pro-inflammatory cytokines (e.g., TNF-α, IFN-γ and IL-1β), indicating that dietary supplementation with 0.3% AMPs could improve anti-inflammatory function in cats. Moreover, stress induces excessive ROS production, which can cause oxidative stress and lipid peroxidation [33]. MDA is the ending product that serves as a marker of lipid peroxidation and oxidative stress, [34] and the levels of antioxidant enzymes, such as SOD, GSH-Px, CAT and T-AOC, can reflect the ability of the organism in eliminating free radicals [35]. In the present study, we found that AMPs increased the antioxidant activities in cats by upregulating GSH-Px and SOD. Consistent with previous studies, we found that compared with the CON group, the concentration of SOD increased in the AMPs group [33,36], which indicates that dietary supplements with AMPs at 0.3% protected cats from oxidative stress induced by transportation.

### 4.2. AMPs Alter Intestinal Microbiota and Metabolite in Ragdoll Cats

Previous studies revealed dietary supplementation with AMPs could regulate gut microbiota, reduce pathogenic bacteria and alter the diversity of microorganisms [22,37,38,39]. In our study, AMPs reduced the richness of bacterial flora; decreased the relative abundances of *Bacteroidetes*, *Proteobacteria*, *Fusobacteria* and *Anaerobiospirillum* on BT1d; and inhibited the growth of *Negativibacillus* on AT1d. Previous studies found that *Bacteroides* are positively correlated with the disease activity index, and *Bacteroidetes* are associated with intestinal inflammation [40,41]. *Proteobacteria* include many pathogenic bacteria, such as *E. coli*, *Salmonella* and *Campylobacter*, which have been associated with dysbiosis and inflammatory disorders [42,43]. *Fusobacteria* was reported to be linked with colon cancer [44]. The genus *Anaerobiospirillum* consists of *Anaerobiospirillum thomasii* and *Anaerobiospirillum succiniciproducens*, which can cause infectious human diseases, such as bacteremia and abdominal infection [45,46]. Previous studies show that the genus *Negativibacillus* is associated with intestinal disorders and many other diseases such as obesity-related disorders and pediatric Crohn’s disease [47,48,49,50]. Our results were further verified with LEfSe analyses. The AMPs group was mainly enriched with beneficial bacteria, including *Eisenbergiella* and *Blautia*. *Eisenbergiella* is the main producer of SCFAs [51]. *Blautia* was reported to alleviate inflammatory diseases and metabolic diseases, and it has potential probiotic functions [52,53]. Collectively, we can infer that AMPs can alleviate the imbalance of microbiota from transportation stress by reducing harmful bacteria and increasing beneficial bacteria.

SCFAs are produced by the colonic bacterial fermentation of non-digestible carbohydrates, and BCFAs are produced by the fermentation of branched-chain amino acids [54]. These microbiota-derived metabolites play important roles in maintaining epithelial function and participate in various metabolic pathways (e.g., lipid and glucose metabolism) [54,55]. In our study, AMPs reduced the fecal acetic acid concentration on BT1d, possibly due to the decreased abundance of *Bacteroides*, which are the main acetic-acid-producing bacteria [56]. However, fecal total BCFAs levels were higher in cats fed with AMPs on AT1d. BCFAs have beneficial effects on preventing intestinal inflammation and increasing the expression of anti-inflammatory cytokines [17]. These results reveal that AMPs might provide protection against transportation-induced inflammation by improving the intestinal’s microbial structure.

Gut microbiota contribute to the host’s metabolism and the regulation of the immune system, thus significantly affecting the host’s physiological functions [57]. We found a high level of serum cholic acid and rifamycin S in the AMPs group. Cholic acid is the primary bile acid, which is synthesized from cholesterol in the liver and reabsorbed by the ileum into the blood’s circulation [58]. Rifamycin S belongs to the ansamycin family, which bears antimicrobial activity [59,60]. The enrichment analysis further revealed that 0.3% AMPs mainly affected lipid metabolism (i.e., bile acid biosynthesis, glycerolipid metabolism and glycerol phosphate shuttle).

Spearman’s correlation analysis found that strong correlations between fecal microbiota and serum metabolites could influence stress parameters, antioxidant capacity and inflammatory responses. *Blautia* was negatively associated with serum phenylethylamine, which was negatively associated with SOD. Furthermore, *Blautia* was positively associated with serum pregnanediol-3-glucuronide, which was negatively associated with diarrhea rates. *Negativibacillus* was negatively associated with serum indoleacrylic acid and indolelactic acid, which were negatively associated with IFN-γ and SAA. The results indicated that phenylethylamine, pregnanediol-3-glucuronide, indoleacrylic acid and indolelactic acid could serve as potential biomarkers of transportation stress. However, the connections between gut microbiota and serum metabolic changes still need to be further explored and validated.

### 4.3. Future Prospects and Drawbacks of the Present Study

AMPs have potential of clinic applications with respect to relieving diarrhea and transportation stress in cats. However, the dosage should be determined more precisely according to the different degrees of symptoms.

In this study, we used ragdoll cats as research subjects partly because the ragdoll cat is a representative and widely owned breed of domestic cats. In addition, a previous study convinced us that the variation between cat breeds is relatively small compared to species such as dogs [61]. However, it is still of interest to carry out studies on other cat breeds in the future to provide more data for clinical use.

## 5. Conclusions

In conclusion, our study indicated that AMPs could beneficially reduce the incidence of diarrhea in cats induced by transportation. In addition, AMPs could decrease the level of inflammatory factors caused by transportation stress, including TNF-α and IL-1β, and enhance the activities of GSH-Px and SOD. Furthermore, AMPs could reduce the abundance of *Negativibacillus* and increase the abundance of *Blautia* and the number of BCFAs. Therefore, the presented data indicated that AMPs supplementation may alleviate diarrhea and oxidative stress in transportation by regulating the gut microbiota and metabolites, thereby supporting gut and host health in cats. In a future study, we will focus on the connection between gut microbiota and metabolites to better understand the precise mechanisms of AMPs on cat health.

## Figures and Tables

**Table 1:** Experimental diet and nutrition composition.

Ingredients	Basis %	Analytical Composition	DM Basis %
Fish	23.00	DM	93.38
Chicken meat meal	24.00	CP	40.17
Duck meat meal	12.00	CF	22.26
Fish meal	8.00	Ash	8.13
Chicken oil	6.00	OM	91.87
Tapioca	5.00		
Tapioca flour	4.00		
Alfalfa grass grain	4.00		
Chicken liver	2.70		
Chicken heart	2.00		
Flavoring paste	1.60		
Salmon oil	1.50		
Beer yeast power	1.20		
Egg power	1.00		
Kelp power	1.00		
Yucca	1.00		
Semen plantaginis	1.00		
Madder	1.00		
Total	100.00		

DM, dry matter; CP, crude protein; CF, crude fat; OM, organic matter.

## Data Availability

The data presented in this study are available in the manuscript.
